# Overexpression of sphingosine-1-phosphate receptor 1 and phospho-signal transducer and activator of transcription 3 is associated with poor prognosis in rituximab-treated diffuse large B-cell lymphomas

**DOI:** 10.1186/1471-2407-14-911

**Published:** 2014-12-03

**Authors:** Jin Ho Paik, Soo Jeong Nam, Tae Min Kim, Dae Seog Heo, Chul-Woo Kim, Yoon Kyung Jeon

**Affiliations:** Tumor Immunity Medical Research Center, Cancer Research Institute, Seoul National University College of Medicine, Seoul, Korea; Department of Pathology, Seoul National University Hospital, 101 Daehak-ro, Jongno-gu, Seoul, 110-744 South Korea; Department of Internal Medicine, Seoul National University College of Medicine, Seoul, Korea; Department of Pathology, Seoul National University Bundang Hospital, Seongnam, Korea

**Keywords:** S1PR1, pSTAT3, Diffuse large B-cell lymphoma, Prognosis

## Abstract

**Background:**

Sphingosine-1-phosphate receptor-1 (S1PR1) and signal transducer and activator of transcription-3 (STAT3) play important roles in immune responses with potential oncogenic roles.

**Methods:**

We analyzed S1PR1/STAT3 pathway activation using immunohistochemistry in rituximab-treated diffuse large B-cell lymphomas (DLBCL; N = 103).

**Results:**

Nuclear expression of pSTAT3 (but not S1PR1) was associated with non-GCB phenotype (p = 0.010). In univariate survival analysis, S1PR1 expression (S1PR1+) was a poor prognostic factor in total DLBCLs (p = 0.018), as well as in nodal (p = 0.041), high-stage (III, IV) (p = 0.002), and high-international prognostic index (IPI; 3–5) (p = 0.014) subgroups, while nuclear expression of pSTAT3 (pSTAT3+) was associated with poor prognosis in the low-stage (I, II) subgroup (p = 0.022). The S1PR1/pSTAT3 risk-categories, containing high-risk (S1PR1+), intermediate-risk (S1PR1-/pSTAT3+), and low-risk (S1PR1-/pSTAT3-), predicted overall survival (p = 0.010). This prognostication tended to be valid in each stage (p = 0.059 in low-stage; p = 0.006 in high-stage) and each IPI subgroups (p = 0.055 [low-IPI]; p = 0.034 [high-IPI]). S1PR1 alone and S1PR1/pSTAT3 risk-category were significant independent prognostic indicators in multivariate analyses incorporating IPI and B symptoms (S1PR1 [p = 0.005; HR = 3.0]; S1PR1/pSTAT3 risk-category [p = 0.019: overall; p = 0.024, HR = 2.7 for S1PR1-/pSTAT3+ vs. S1PR1+; p = 0.021, HR = 3.8 for S1PR1-/pSTAT3- vs. S1PR1+]).

**Conclusions:**

Therefore, S1PR1 and S1PR1/pSTAT3 risk-category may contribute to risk stratification in rituximab-treated DLBCLs, and S1PR1 and STAT3 might be therapeutic targets for DLBCL.

## Background

Diffuse large B-cell lymphoma (DLBCL) is a biologically and clinically heterogeneous entity that accounts for 30-50% of non-Hodgkin lymphomas, depending on geographical area [1, 2]. Germinal center B-cell-like (GCB) and activated B-cell-like (ABC)/non-GCB subgroups were previously identified as two distinct subgroups of DLBCL that showed differentially activated signaling pathways [1, 3–6]. Typically, the NF-κB pathway is constitutively activated in ABC-like DLBCLs and cooperates with the STAT3 pathway to promote cell survival [7–9], while dependency on the PI3K/Akt pathway has been demonstrated in GCB-type DLBCL [10]. Recently, high-throughput techniques have revealed more complex features of genetic alterations and identified novel therapeutic pathways in DLBCL [9, 11, 12]. One of the promising candidate pathways for targeted therapy in DLBCL is the STAT3 pathway [13].

Unlike inflammatory conditions with transient STAT3 activation, STAT3 is aberrantly and constitutively activated in many cancers, including hematolymphoid malignancies [14]. Activated STAT3, *i.e.,* phospho-STAT3 (pSTAT3), is transported into the nucleus, functioning as a transcription factor for various genes involving cellular apoptosis, proliferation, and survival [15]. In lymphomas, the expression and activation of STAT3 have previously been investigated in human lymphoma tissues and cell lines [16–18]. The nuclear expression of STAT3 or pSTAT3 alone, as detected by immunohistochemistry, was shown to be a poor prognostic factor in all DLBCL patients, including the GCB and non-GCB/ABC subgroups [16, 17].

S1PR1 is a member of the G-protein-coupled receptor for sphingosine-1-phosphate (S1P), a chemokine mediating immune cell migration [19, 20]. S1P is produced intracellularly by sphingosine kinase (SPHK) 1/2; it is released from the cells and then binds to the S1P receptors (S1PR1-S1PR5) of target cells in an autocrine and/or paracrine manner [20, 21]. S1PR1 transduces intracellular signals, leading to various biologic effects, including cell proliferation, survival and migration via the ERK, Akt, and Rac pathways, respectively. Recently, it has been reported that *S1PR1* is also transcribed by pSTAT3, and enhanced S1PR1 subsequently and reciprocally activates STAT3, thus building a positive feedback loop that involves the S1PR1/pSTAT3 pathway, which is important for consistent STAT3 activation in mouse and human solid tumors and tumor-associated myeloid cells [22].

At present, only a few studies on S1PR1 in malignant lymphoma are available. Hodgkin lymphoma and mantle cell lymphoma showed S1PR1 expression in cell lines or tissues, suggesting potential biologic roles for S1PR1 in this context [23, 24]. Furthermore, co-activation of S1PR1 and STAT3 was observed in ABC-DLBCL cells and tissues, and S1PR1 was suggested as a potential target for blocking STAT3 activation [18]. However, there have been no integrated studies on the clinicopathologic and prognostic implications of S1PR1 and STAT3 activation in DLBCL patients. We hypothesized that S1PR1, STAT3, and/or the co-activation of S1PR1/STAT3 pathway might be useful prognostic markers in DLBCL. In this study, we comprehensively investigated the expression of S1PR1 and pSTAT3 and analyzed their correlation with clinicopathologic features and impacts on clinical outcomes in rituximab-treated DLBCL patients.

## Methods

### Patients

A total of 103 patients, who were diagnosed with DLBCL at Seoul National University Hospital from 2001 to 2010 and treated with rituximab-based chemotherapy, were enrolled. The patients’ histologic slides and clinical medical records were reviewed by two experienced hemato-pathologists (J.H.P. and Y.K.J.) and hemato-oncologists (T.M.K. and D.S.H), respectively. The follow-up duration ranged from 0 to 105 months (median, 22 months). In total, 32 patients (31%) had died at the time of analysis. The Institutional Review Board of Seoul National University Hospital approved this study (1012-053-344). Informed consent for participation in the study was waivered by the Institutional Review Board of Seoul National University Hospital on the basis that this study was a retrospective study using archived material, and did not increase risk to the patients.

### Immunohistochemistry and classification of germinal center B-cell (GCB) and non-GCB phenotype DLBCL

For immunohistochemistry (IHC), 2-mm-diameter cores were taken from representative formalin-fixed paraffin-embedded (FFPE) tissue blocks of patients, and tissue microarrays (TMAs) were manufactured as previously described [25]. DLBCL was classified into GCB and non-GCB phenotypes on the basis of the Hans and Choi classifications with CD10, bcl-6, MUM1, GCET1 and FoxP1 immunostaining, as previously described [3, 4, 25].

IHC for S1PR1 and pSTAT3 were performed using the Leica BOND-MAX automated immunostainer (Leica Microsystems, Wetzlar, Germany) and the following antibodies: S1PR1 (rabbit polyclonal, EDG-1 (H60), Santa Cruz, Dallas, TX, USA) and pSTAT3 (Y705) (D3A7, rabbit monoclonal, Cell Signaling, Danvers, MA, USA). Consensus interpretation of IHC was performed by two hematopathologists (J.H.P. and Y.K.J.) using multi-head light microscope (BX43, Olympus, Tokyo, Japan) for one core per each case.

Of the 103 DLBCLs, immunophenotyping was successful in 99 cases. Of them, 8% (8/99) were discordant between the Hans and Choi classifications. Specifically, 5 cases were classified as GCB by Hans but ABC by Choi, whereas 3 cases were classified as non-GCB by Hans but GCB by Choi. The remaining 91 cases were classified concordantly, with 32 classified as GCB and 59 classified as non-GCB/ABC. Because no clinicopathologic differences were observed between subtypes according to the Hans or Choi classification and S1PR1/pSTAT3 expression, in this study, further analysis was performed using the Hans classification.

### Statistical analysis

Statistical analysis was performed using SPSS 18.0 (SPSS Inc., Chicago, IL, USA). Cross-table analysis was performed using a two-sided Pearson’s χ^2^-test. Survival analysis was performed using Kaplan-Meier (univariate) and Cox proportional hazard models (multivariate) for overall survival. P-values <0.05 were considered statistically significant.

## Results

### Clinical features of rituximab-treated DLBCL

As shown in Table 1, the median age of patients was 61 years and ranged from 14 to 79 years. Patients with male sex (59%, 59/99), primary extranodal disease (64%, 64/99), and low international prognostic index (IPI; score 0–2; 62%, 57/92) were more frequent. All patients were treated with rituximab-containing regimens. In 94% (94/99), the patients were treated with rituximab, cyclophosphamide, doxorubicin, vincristine, and prednisolone (R-CHOP). There were no significant differences in clinical variables between the GCB and non-GCB phenotypes.

**Table 1:** **Clinicopathologic features of rituximab-treated diffuse large B cell lymphoma patients (N = 103)**

Variables	Total n (%)	GCB ^*^ n (%)	Non-GCB ^*^ n (%)
Age, years			
Mean (range)	57.7 (14–79)	60.5 (23–79)	56.2 (14–78)
<60	50 (49%)	13 (35%)	35 (56%)
≥60	53 (51%)	24 (65%)	27 (44%)
Sex			
Male	61 (59%)	23 (62%)	36 (58%)
Female	42 (41%)	14 (38%)	26 (42%)
Primary site			
Nodal	37 (36%)	14 (38%)	21 (34%)
Extranodal	66 (64%)	23 (62%)	41 (66%)
Ann Arbor stage			
I, II	47 (46%)	19 (51%)	27 (44%)
III, IV	56 (54%)	18 (49%)	35 (56%)
IPI group^†^			
Low (0–2)	60 (63%)	24 (65%)	33 (60%)
High (3–5)	36 (37%)	13 (35%)	22 (40%)
B symptoms^†^			
Absent	74 (76%)	31 (84%)	43 (72%)
Present	24 (24%)	6 (16%)	17 (28%)
ECOG PS^†^			
0, 1	86 (84%)	34 (92%)	49 (80%)
≥2	16 (16%)	3 (8%)	12 (20%)
LDH†			
Normal	43 (45%)	14 (39%)	26 (46%)
Elevated	53 (55%)	22 (61%)	30 (54%)
BM involvement^†^			
Absent	85 (86%)	34 (92%)	48 (83%)
Present	14 (14%)	3 (8%)	10 (17%)
Number of extranodal sites			
0, 1	75 (73%)	26 (70%)	46 (74%)
≥2	28 (27%)	11 (30%)	16 (26%)
EBER^†^			
Negative	95 (94%)	37 (100%)	55 (90%)
Positive	6 (6%)	0 (0%)	6 (10%)
Treatment			
Rituximab + CHOP	97 (94%)	37 (100%)	57 (92%)
Rituximab + others	6 (6%)	0 (%)	5 (8%)

GCB, germinal center B-cell like; IPI, international prognostic index; ECOG PS, Eastern Cooperative Oncology Group performance status; LDH, lactate dehydrogenase; BM, bone marrow; EBER, EBV-encoded RNA; CHOP, cyclophosphamide, doxorubicin, vincristine, prednisolone. ^*^GCB and non-GCB phenotypes were classified using Hans classification with four unclassifiable cases. ^†^The number excludes missing values.

### Expression patterns of S1PR1 and pSTAT3 in DLBCLs

**Figure 1:**
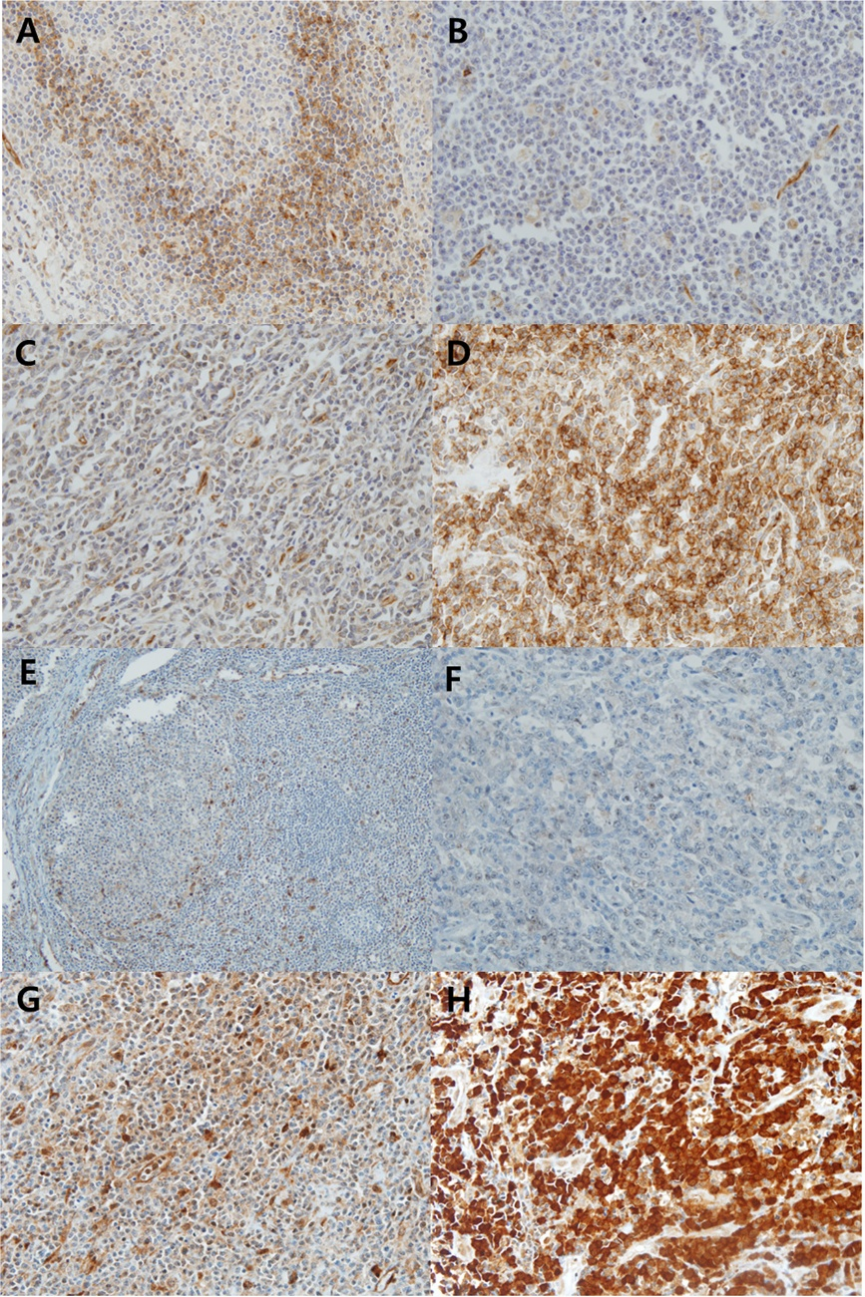
**S1PR1 and pSTAT3 immunostaining patterns. (A)** Reactive mantle zone B-cells and endothelial cells express S1PR1. S1PR1 immunostaining was considered to be negative for cases with no staining **(B)** or weaker staining than mantle zone B-cells **(C)** and positive for cases with similar to or stronger staining than mantle zone B-cells **(D)**. **(E)** Histiocytes and endothelial cells are stained for pSTAT3. pSTAT3 immunostaining was considered negative for cases with no staining **(F)** or cytoplasmic staining **(G)** and positive for the cases with nuclear staining in tumor cells **(H)**.

Consistent with a previous report [24], S1PR1 was expressed in the cytoplasm of reactive mantle zone B-cells and endothelial cells in non-neoplastic tonsils (Figure 1A). In DLBCLs, S1PR1 was stained in the cytoplasm of tumor cells with variable intensities and proportions (Figure 1B-D). Cases showing S1PR1 staining with an intensity similar to or stronger than reactive mantle B-cells in more than 30% of tumor cells were interpreted as being positive for S1PR1 expression. pSTAT3 was stained in the histiocytes and endothelial cells of non-neoplastic tonsils (Figure 1E). In DLBCLs, pSTAT3 was stained in the cytoplasm or in both the cytoplasm and nucleus (Figure 1F-H). Considering that the active form of STAT3 (pSTAT3) is transported into the nucleus to be functional [14], cases that exhibited nuclear staining in more than 30% of tumor cells were interpreted as being positive for pSTAT3 expression. Using these criteria, S1PR1 expression was positive in 40% (41/103) and pSTAT3 expression was positive in 59% (61/103) of DLBCL (Table 2).

**Table 2:** **Correlation between S1PR1/pSTAT3 expression and clinicopathologic variables**

variables	S1PR1				pSTAT3			
	Negative	Positive	Total	P ^*^	Negative	Positive	Total	P ^*^
Age, years								
Mean (range)								
<60	30 (48%)	20 (49%)	50 (49%)	0.969	21 (50%)	29 (48%)	50 (49%)	0.806
≥60	32 (52%)	21 (51%)	53 (51%)		21 (50%)	32 (52%)	53 (51%)	
Sex								
Male	34 (55%)	29 (67%)	61 (59%)	0.265	28 (67%)	33 (54%)	61 (59%)	0.202
Female	28 (45%)	14 (33%)	42 (41%)		14 (33%)	28 (46%)	42 (41%)	
Primary nodal disease								
Nodal	27 (44%)	10 (24%)	37 (36%)	0.047^†^	21 (50%)	16 (26%)	37 (36%)	0.013^†^
Extranodal	35 (56%)	31 (76%)	66 (64%)		21 (50%)	45 (74%)	66 (64%)	
Primary UAT disease								
Non-UAT	55 (89%)	28 (68%)	83 (81%)	0.010^†^	39 (93%)	44 (72%)	83 (81%)	0.009^†^
UAT	7 (11%)	13 (32%)	20 (19%)		3 (7%)	17 (28%)	20 (19%)	
Ann Arbor stage								
I, II	26 (42%)	21 (51%)	47 (46%)	0.354	18 (43%)	29 (48%)	47 (46%)	0.639
III, IV	36 (58%)	20 (49%)	56 (54%)		24 (57%)	32 (52%)	56 (54%)	
IPI group^‡^								
Low (0–2)	36 (61%)	24 (65%)	60 (63%)	0.705	23 (59%)	37 (65%)	60 (63%)	0.555
High (3–5)	23 (39%)	13 (35%)	36 (37%)		16 (41%)	20 (35%)	36 (37%)	
B symptoms^‡^								
Absent	46 (76%)	31 (78%)	77 (76%)	0.809	36 (86%)	41 (69%)	77 (76%)	0.059
Present	15 (24%)	9 (22%)	24 (24%)		6 (14%)	18 (31%)	24 (24%)	
ECOG PS^‡^								
0, 1	54 (87%)	32 (80%)	86 (84%)	0.336	36 (86%)	50 (83%)	86 (84%)	0.745
≥2	8 (13%)	8 (20%)	16 (16%)		6 (14%)	10 (17%)	16 (16%)	
LDH^‡^								
Normal	25 (43%)	18 (47%)	43 (45%)	0.681	16 (42%)	27 (47%)	43 (45%)	0.668
Elevated	33 (57%)	20 (53%)	53 (55%)		22 (58%)	31 (53%)	53 (55%)	
BM involvement^‡^								
Absent	56 (90%)	29 (78%)	85 (86%)	0.099	38 (95%)	47 (80%)	85 (86%)	0.032^†^
Present	6 (10%)	8 (22%)	14 (14%)		2 (5%)	12 (20%)	14 (14%)	
Number of extranodal sites								
0, 1	45 (73%)	30 (73%)	75 (73%)	0.947	30 (71%)	45 (74%)	75 (73%)	0.793
≥2	17 (27%)	11 (27%)	28 (27%)		12 (29%)	16 (26%)	28 (27%)	
EBER^‡^								
Negative	58 (94%)	37 (95%)	95 (94%)	0.784	39 (93%)	56 (95%)	95 (94%)	0.666
Positive	4 (6%)	2 (5%)	6 (6%)		3 (7%)	3 (5%)	6 (6%)	
Hans classification^‡^								
GCB	24 (40%)	13 (33%)	37 (37%)	0.503	21 (53%)	16 (27%)	37 (37%)	0.010^†^
Non-GCB	36 (60%)	26 (67%)	62 (63%)		19 (47%)	43 (73%)	62 (63%)	
pSTAT3 nuclear expression								
Negative	28 (45%)	14 (34%)	42 (41%)	0.265	-	-	-	-
Positive	34 (55%)	27 (66%)	61 (59%)		-	-	-	
Total	62 (100%)	41 (100%)	103 (100%)		42 (100%)	61 (100%)	103 (100%)	

DLBCL, diffuse large B-cell lymphoma; GCB, germinal center B-cell like; ABC, activated B-cell like; IPI, international prognostic index; ECOG PS, Eastern Cooperative Oncology Group performance status; LDH, lactate dehydrogenase; BM, bone marrow; EBER, EBV-encoded RNA; UAT, upper aerodigestive tract. ^*^P values were calculated using Pearson’s chi-square test. ^†^indicates P values are less than 0.05. ^‡^The number excludes missing values.

### Relationships between S1PR1/pSTAT3 expression and clinicopathologic variables

The correlations between S1PR1/pSTAT3 expression and clinicopathologic features are summarized in Table 2. Briefly, the expression of S1PR1 and pSTAT3 was more frequently observed in DLBCLs, primarily occurring in extranodal sites (p = 0.047 for S1PR1; p = 0.013 for pSTAT3) and upper aerodigestive tract (UAT) including nasal cavity, nasopharynx, oral cavity, oropharynx, and hypopharynx (p = 0.010 for S1PR1; p = 0.009 for pSTAT3). Bone marrow involvement was more common in patients with pSTAT3-positive DLBCLs (p = 0.032). Otherwise, no significant relationships were observed between the expression of S1PR1/pSTAT3 and other clinical variables. pSTAT3 expression was much more frequently observed in non-GCB cases than in GCB cases (p = 0.010), while S1PR1 expression was not significantly different between these groups.

### Univariate survival analysis with conventional clinicopathologic variables and S1PR1/pSTAT3

**Figure 2:**
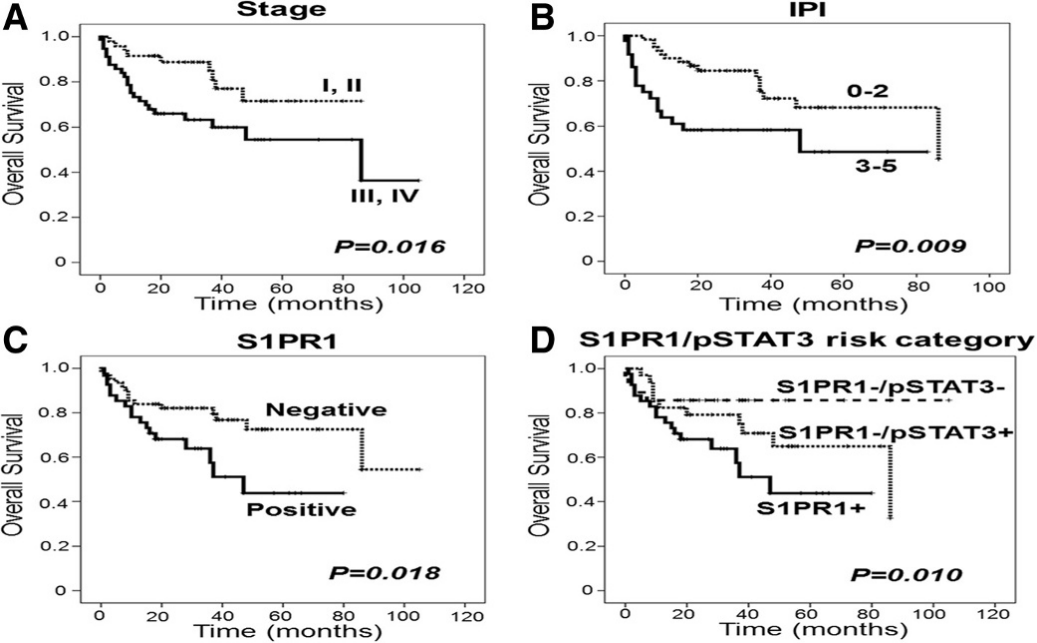
**Kaplan-Meier survival curves for overall survival with log-rank test. (A)** Stage, **(B)** international prognostic index, **(C)** S1PR1, and **(D)** S1PR1/pSTAT3 risk category were significant prognostic factors in rituximab-treated diffuse large B-cell lymphoma patients.

As shown in Table 3, univariate survival analysis for overall survival was performed in a total of 103 cases of DLBCL, as well as in the following subgroups: nodal vs. extranodal, low stage (I, II) vs. high stage (III, IV), low IPI (0–2) vs. high IPI (3–5), and GCB vs. non-GCB. In the total cohort, high stage and high IPI were significant poor prognostic factors (p = 0.016 for stage; p = 0.009 for IPI; Figure 2A and B), along with old age (>60 y), presence of B symptoms, and high LDH level (Table 3).

**Table 3:** **Univariate survival analysis of S1PR1/pSTAT3 expression and clinicopathologic variables for overall survival in rituximab-treated DLBCL patients (total cohort) and clinicopathologic subgroups**

Clinicopathologic variables	P values in each group ^*^ by univariate analysis								
	Rituximab –treated DLBCL (total cohort) (N =103)	Primary site		Stage		IPI†		Hans classification†	
		Nodal subgroup (n = 37)	Extranodal (n = 66)	Low stage (n = 47)	High stage (n = 56)	Low (0–2) (n = 60 )	High (3–5) (n = 36 )	GCB (n = 37)	Non-GCB (n = 62)
Age >60	0.019	0.027	NS (0.239)	NS (0.050)	NS (0.223)	NS (0.141)	NS (0.391)	NS (0.052)	0.036
Stage (III, IV)	0.016	0.034	NS (0.145)	NA	NA	NS (0.147)	NS (0.110)	NS (0.652)	NS (0.008)
High IPI (3–5)	0.009	0.039	NS (0.087)	<0.001	0.339	NA	NA	NS (0.104)	0.047
B symptoms	0.014	NS (0.576)	0.002	NS (0.481)	0.038	NS (0.836)	NS (0.052)	NS (0.569)	0012
High LDH	0.016	NS (0.078)	NS (0.063)	NS (0.083)	NS (0.376)	NS (0.585)	NS (0.157)	NS (0.955)	0.002
BM involvement	NS (0.663)	NS (0.790)	NS (0.323)	NS	NS (0.711)	NS (0.418)	NS (0.362)	NS (0.326)	NS (0.478)
No. of extranodal sites	NS (0.959)	NS (0.175)	NS (0.556)	NS	NS (0.116)	NS (0.787)	0.026	NS (0.701)	NS (0.662)
ECOG PS	NS (0.115)	NS (0.528)	NS (0.147)	NS (0.264)	NS (0.540)	NS (0.531)	NS (0.679)	0.006	NS (0.756)
Hans classification	NS (0.764)	NS (0.118)	NS (0.429)	NS (0.336)	NS (0.404)	NS (0.789)	NS (0.709)	NA	NA
S1PR1	0.018	0.041	NS (0.127)	NS (0.880)	0.002	NS (0.347)	0.014	NS (0.092)	NS (0.238)
pSTAT3	NS (0.713)	NS (0.536)	NS (0.996)	0.022	NS (0.267)	NS (0.067)	NS (0.248)	NS (0.143)	NS (0.256)
S1PR1/pSTAT3 risk category	0.010	NS (0.079)	NS (0.254)	NS (0.059)	0.006	NS (0.055)	0.034	NS (0.136)	NS (0.498)

NS, not significant; NA, not applicable; GCB, germinal center B-cell-like; ABC, activated B-cell-like; DLBCL, diffuse large B-cell lymphoma; IPI, international prognostic index; ECOG PS, Eastern Cooperative Oncology Group performance status; LDH, lactate dehydrogenase; BM, bone marrow. ^*^P values less than 0.05 are considered significant by Kaplan-Meier univariate analysis for overall survival. ^†^The number excludes missing values.

The expression of S1PR1 was a significant poor prognostic factor (p = 0.018; Figure 2C), while pSTAT3 expression was not. However, in the subgroup analyses (Table 3), S1PR1 was a significant poor prognostic factor in the nodal (p = 0.041), high stage (p = 0.002), and high IPI subgroups (p = 0.014). In contrast, pSTAT3 expression was associated with shorter overall survival in patients with low stage (p = 0.022) and low IPI group (p = 0.067).

### Survival analysis with risk stratification model using S1PR1/pSTAT3

Given the integrated role of S1PR1 and pSTAT3 in oncogenesis [22] and the above observation that suggested a possible complementary effect of S1PR1 and pSTAT3 on the prognosis of DLBCLs, we made a new immunohistochemical variable combining S1PR1 and pSTAT3 expression, specifically, a S1PR1/pSTAT3 risk category. In brief, this S1PR1/pSTAT3 risk category was useful for predicting the prognosis of DLBCL patients who were defined as follows: 1) high risk: S1PR1+, 2) intermediate risk: S1PR1-/pSTAT3+, and 3) low risk group: S1PR1-/pSTAT3-. The S1PR1/pSTAT3 risk category was a significant prognostic factor in the total cohort of rituximab-treated DLBCL patients (N = 103) (Figure 2D). Furthermore, this risk category tended to be valid in low stage (p = 0.059) and high stage (p = 0.006) subgroups, as well as in the low IPI (p = 0.055) and high IPI subgroups (p = 0.034) (Table 3).

### Multivariate survival analysis with conventional clinicopathologic variables, S1PR1 and S1PR1/pSTAT3 risk category

To further determine the prognostic implication of S1PR1 and pSTAT3 expression, multivariate survival analysis was performed in the total cohort of rituximab-treated DLBCL incorporating the S1PR1 or S1PR1/pSTAT3 risk category and conventional prognostic variables (Table 4).

**Table 4:** **Multivariate survival analysis of S1PR1/pSTAT3 expression and clinicopathologic variables for overall survival in a total cohort of rituximab-treated DLBCL patients (N = 103)**

**Multivariate analysis with IPI, B symptoms, and S1PR1**				
**Clinicopathologic variables**	**Category**	**Univariate analysis**	**Multivariate analysis**	
		**P** ^*^	**P** ^*^	**HR [95% CI]**
IPI	3-5 vs. 0-2	0.009	0.019	2.7 [1.2-6.2]
B symptoms	present vs. absent	0.014	0.180	1.7 [0.8-3.9]
S1PR1	positive vs. negative	0.018	0.005	3.0 [1.4-6.5]
**Multivariate analysis with IPI, B symptoms, and S1PR1/pSTAT3 risk category**				
**Clinicopathologic variables**	**Category**	**Univariate analysis**	**Multivariate analysis**	
		**P** ^*^	**P** ^*^	**HR [95% CI]**
IPI	3-5 vs. 0-2	0.009	0.021	2.7 [1.2-6.2]
B symptoms	present vs. absent	0.014	0.208	1.6 [0.7-3.9]
S1PR1/pSTAT3 risk category		0.010	0.019	
	high risk (S1PR1+) vs. intermediate risk (S1PR1-/pSTAT3+)		0.024	2.7 [1.1-6.2]
	high risk (S1PR1+) vs. low risk (S1PR1-/pSTAT3-)		0.021	3.8 [1.2-11.6]

DLBCL, diffuse large B-cell lymphoma; IPI, international prognostic index. ^*^P values less than 0.05 are considered significant.

In the multivariate Cox analysis with IPI, B symptoms and S1PR1, IPI and S1PR1 were independent prognostic factors for overall survival (p = 0.019, hazard ratio [HR] = 2.7 for IPI; p = 0.005, HR = 3.0 for S1PR1). When the S1PR1/pSTAT3 risk category was included in the multivariate modeling, IPI and S1PR1/pSTAT3 risk category were also found to be independent prognostic factors (p = 0.021, HR = 2.7 for IPI; p = 0.019 for S1PR1/pSTAT3 risk category; p = 0.024, HR = 2.7 for high risk [S1PR1+] vs. intermediate risk [S1PR1-/pSTAT3+]; p = 0.021, HR = 3.8 for high risk [S1PR1+] vs. low risk [S1PR1-/pSTAT3-]) (Table 4). Together, these data demonstrate that both S1PR1 expression and S1PR1/pSTAT3 risk category are independent prognostic predictors in DLBCL patients treated with rituximab-based chemotherapy.

## Discussion

In the present study, we demonstrated for the first time that 1) S1PR1 is an independent prognostic factor in rituximab-treated DLBCL patients, and 2) the S1PR1/pSTAT3 risk category is useful for risk stratification of DLBCL patients.

Given that S1PR1 and pSTAT3 closely co-operate during inflammatory and immunologic processes and neoplasms, the S1PR1/pSTAT3 risk category was developed to help stratify the risk of rituximab-treated DLBCL patients using immunohistochemistry for S1PR1 and pSTAT3. This category has prognostic value in a stage- and IPI-independent manner. Notably, DLBCLs with S1PR1 expression exhibited worst prognosis regardless of pSTAT3 expression and represented the high risk (S1PR1+) group. Meanwhile, DLBCLs without S1PR1 expression could be divided into a low risk group (S1PR1-/pSTAT3-), which is assumed to have an inactive form of S1PR1 and pSTAT3, and an intermediate risk group (S1PR1-/pSTAT3+), which might reflect pSTAT3 activation via an alternative non-S1PR1 pathway. These data suggest that aside from the S1PR1/pSTAT3 positive feedback loop, S1PR1- or pSTAT3-associated alternative signaling pathways might also be involved in the biology of DLBCL. In fact, in the present study, S1PR1 and pSTAT3 expression did not correlate significantly with each other (p = 0.265). Because S1P-S1PR1 signaling is known to be associated with several important pathways, including the mTOR pathway in T cells [26] and the Akt pathway in non-lymphoid cells [27, 28], it is possible that S1PR1-mediated oncogenic signaling pathways other than STAT3 might underlie the aggressive behavior of S1PR1-positive DLBCLs. As such, the possible roles of S1PR1 in the biology of DLBCLs via mechanisms other than STAT3 signaling remain to be clarified.

It was previously reported that STAT3 or pSTAT3 was a prognostic factor in DLBCLs [16, 17]. In Wu’s series of 74 patients, approximately half of whom had been treated with R-CHOP therapy, the prognosticator was not pSTAT3, but STAT3, although the expression of these two molecules was highly concordant [16]. STAT3 activation has previously been described as a main mechanism of ABC-DLBCL [18], and in Huang’s series of 185 patients who had undergone R-CHOP therapy, pSTAT3 was a significant prognostic factor for event-free survival in ABC-DLBCL [17]. In the present study, the nuclear expression of pSTAT3 was much higher in the non-GCB type and tended to be associated with shorter overall survival in patients in the early stages or in those who had low IPIs. However, overall, pSTAT3 expression alone was not an independent prognostic factor in DLBCL patients treated with rituximab and showed no prognostic significance in either the GCB or non-GCB/ABC subgroup. Based on these observations, pSTAT3 expression itself is thought to have an influence on the prognosis of DLBCLs in a more complicated way, which remains to be further investigated.

S1PR1 signaling can be blocked by an effective inhibitor, FTY720, which was developed as an immunosuppressant and is being used in the treatment of patients with multiple sclerosis [20]. Moreover, other selective S1PR1 modulators such as Syl930 have been developed to reduce side effects including bradycardia [29]. These molecules may be useful for functional studies to clarify the role of S1PR1 signaling in the biology of DLBCL. In a previous study by Liu, STAT3 was co-localized with S1PR1 in the tumor cells of a few ABC DLBCL tissues, and the inhibition of S1PR1 with FTY720 or *S1PR1* shRNA successfully suppressed STAT3 activity and tumor cell growth in vitro and in an in vivo murine lymphoma model [18]. SPHK1, which catalyzes S1P production within the cells, was more frequently expressed in B-cell non-Hodgkin lymphomas with higher clinical grade [30]. Considering that the immunohistochemical expression of S1PR1 was independently associated with poor clinical outcome of DLBCL patients in the present study, the S1P/S1PR1 pathway is suspected to play a role in contributing to the aggressive behavior of DLBCL and, in addition to STAT3, is considered to be a promising therapeutic target in DLBCL.

To measure the expression level of proteins in this study, we used IHC method and graded according to the intensities and proportions of the stained tumor cells in DLBCL tissues. To more validate our data, a proteomic study using spectral abundance in a shotgun study or a more quantitative multiple reaction monitoring might also be applicable in future studies [31, 32].

Another notable finding in this study is that S1PR1 and pSTAT3 are frequently expressed by DLBCLs primarily occurring in the extranodal and UAT areas. Although UAT has been described as a unique site of extranodal NK/T cell lymphoma, no specific features have been recognized for DLBCLs of the primary UAT lesion. Interestingly, a recent study revealed that S1P/S1PR1/STAT3 signaling was an important link between chronic intestinal inflammation and colitis-associated cancer [33]. Considering the important role of the S1PR1/STAT3 pathway in the inflammatory reaction and inflammation-associated carcinogenesis, this anatomic predilection of S1PR1 and STAT3-expressing DLBCLs suggests that the S1PR1/STAT3 pathway may be involved in lymphomagenesis in inflammation-prone areas, such as UAT.

## Conclusions

We demonstrate here that S1PR1 is a new immunohistochemical prognostic marker and that the S1PR1/pSTAT3 risk category can be used for risk stratification in rituximab-treated DLBCL patients. We also suggest that the S1PR1 may be a potential therapeutic target in a subset of DLBCLs.
